# The Effect of Municipal Biosolids on the Growth, Physiology and Synthesis of Phenolic Compounds in *Ocimum basilicum* L.

**DOI:** 10.3390/ijms25010448

**Published:** 2023-12-28

**Authors:** Andrei Lobiuc, Vasile Stoleru, Roxana Gheorghiţă, Marian Burducea

**Affiliations:** 1 Department of Medicine and Biological Sciences, Stefan cel Mare University, 720229 Suceava, Romania; andrei.lobiuc@usm.ro (A.L.); roxana.puscaselu@usm.ro (R.G.); 2 Department of Horticulture Technologies, “Ion Ionescu de la Brad” University of Life Sciences, 700490 Iasi, Romania; vstoleru@uaiasi.ro; 3 Research and Development Station for Aquaculture and Aquatic Ecology, “Alexandru Ioan Cuza” University, Carol I, 20A, 700505 Iasi, Romania

**Keywords:** cultivation substrates, basil, photosynthesis, chlorophyll fluorescence, antioxidant enzyme, antioxidant activity, polyphenols, rosmarinic acid

## Abstract

The continuous development of drinking water networks is leading to the production of increasing amounts of waste water and sewage sludge. Secondary-treated sewage sludge is called biosolids and can be used as fertilizers in agriculture due to its rich nutrient content. The aim of this study was to evaluate the effects of biosolids mixed with an eroded soil on the morphology, physiology and synthesis of bioactive compounds in basil. The study was performed in pots under laboratory-controlled conditions. In total, four substrates were tested: S1 biosolids 100%, S2 biosolids 15% + eroded soil 85%, S3 eroded soil 100% and S4 control (commercial growing substrate). At the morphological level, a significant increase in plant height, number of branches, fresh biomass and dry biomass was found in the S2 variant. At the physiological level, photosynthesis and chlorophyll content did not vary significantly, but the quantum yield of PSII (ΦPSII) was significantly higher at S1 and S2. The oxidative status evaluated by determining the activity of SOD, POD and CAT enzymes was better in S2 and S3 compared to S3. Regarding the synthesis of bioactive compounds (rosmarinic acid, caffeic acid and gallic acid), it was stimulated in S1 and S2. In conclusion, biosolids application stimulated the stress response mechanisms in basil plants by increasing the quantum yield chlorophyll fluorescence and catalase activity, alleviating the negative effects of eroded soil.

## 1. Introduction

Ensuring access to potable water for the entire population is one of the most important Sustainable Development Goals and is a political priority in the European Union (EU). However, the continuous development of drinking water networks is leading to the production of increasing amounts of waste water. Following the treatment process of waste water, sewage sludge results [1]. At the level of the European Union (EU), approximately 10 million tons of dry sewage sludge are produced annually, and this amount will continue to increase with the development of drinking water networks in rural areas of countries such as Romania [2]. These high amounts require an optimal disposal strategy that takes into account the environmental impact, economic costs and public perception [3]. Currently, the main destination of sewage sludge in the EU is agriculture, where approximately 49.2% is used as fertilizer [4]. According to Eurostat data from 2017, the largest sewage-sludge-producing countries in the EU are Germany, Spain and France with 1785.55, 1192.00, 1174.00 thousand tonnes (t) dry substance (d.s.) per year (yr), of which, the largest part is used in agriculture (311.99, 997.10, 299.00 thosand t d.s.). By comparison, Romania produced 283.34 thousand t d.s., of which, only 35 thousand t d.s. were used in agriculture, which highlights the potential for expanding the use of sewage sludge in Romanian agriculture [5]. In order to comply with the legislative norms regarding the use in agriculture (Sewage Sludge Directive 86/278/EEC), the sewage sludge is treated secondary to eliminate the risk of contamination with pollutants. The secondary-treated sewage sludge is called biosolids. The relatively high content of heavy metals in biosolids has led some researchers to monitor their transfer from the substrate to the plant and edible organs. For example, Elmi et al., 2019 [6] tested the biosolids from a water treatment plant in Kuwait on tomatoes and showed that Cd and Pb are not found in the tomato fruits, and the transfer factors from the substrate to the root were Zn > Cu > Cr > Cd and in fruits Zn > Cu > Cr, suggesting that the risk of heavy metal transfer to tomato fruits is minimal. The response of plants to fertilization with biosolids varies depending on the application method (on the surface or embedded in the soil), the chemical composition of the biosolids and the soil, the application dose and the cultivated species [1]. Application of biosolids as fertilizers contributes to the improvement in physicochemical properties of the soil by increasing the content of organic matter and nutrients (total nitrogen, mobile forms of phosphorus and potassium), increasing the water retention capacity, increasing the air spaces and reducing the density [7].

Soil erosion is a global problem that affects agricultural production capacity endangering food security in the context of population growth. The magnitude of this phenomenon is very high, being estimated at 35.9 billion tonnes per year for 2012, increasing by 2.5% in comparison to the level measured in 2001, mainly due to the change in land use destination [7]. The causes that lead to soil erosion are both natural, for example, erosion caused by rain and wind, and anthropogenic caused by deforestation or intensive agriculture. Soils affected by erosion lose nutrients through runoff, a phenomenon with a high economic impact estimated at USD 400 billion per year [8]. Recent modeling has shown that at the European level approximately 25% of the surface is affected by erosion above the recommended sustainability level of 2 t/ha/yr, and over 6% of agricultural lands suffer from severe erosion (11 t/ha/yr) [9]. In Romania, soil erosion caused by precipitation is 2.84 t/ha/yr, exceeding the EU average of 2.46 t/ha/yr [10]. More than 50% of the arable land is affected by erosion, the amplitude of this phenomenon reaching 30–45 t/ha/year in the Moldavian Plateau [11]. Moreover, approximately 4.8 million ha have a low and very low content of organic matter [12]. The negative effects of erosion on soil used for agriculture are represented by the alteration in soil functions for crop growth such as the supply of water, nutrients and rooting space. Moreover, soil microorganism communities are affected due to the degradation of environmental conditions, effects manifested by the reduction of their diversity and biomes. These conditions determine the appearance of stress in cultivated plants affecting the germination of seeds, the growth and development process, and ultimately the crop yield [13,14]. Soil erosion affects the development of the plant root system by the mechanics of geomorphological processes and result in the reduction of root-to-shoot ratios. At the physiological level, the main negative effect is caused by the reduced or lack of water leading to photosynthesis reduction [15]. Furthermore, the rate of photosynthesis is also limited by the decrease in Rubisco or Rubp content in the leaf, given the reduced content of N in the eroded soil. The application of conservative management measures can reduce both the soil erosion process and its negative effects, restoring productivity and ecosystem services. The application of biosolids can be beneficial in the case of soils affected by erosion through the high supply of organic matter and nutrients. Also, biosolids can contribute to improving the physical properties of the soil by increasing the air spaces, increasing the water retention capacity, and decreasing the degree of compaction. At the same time, taking into account the peculiarities of eroded soils, the slope of the land and the degree of transport of eroded particles, laboratory and open field studies are necessary to be able to answer questions such as: Does the administration of biosolids on lands affected by erosion lead to the stabilization of these soils? Can the fertilization of eroded soils with biosolids be considered a conservative method with a soil protection role? Based on the above considerations, the objective of this study was to evaluate the effects of basil cultivation on a substrate consisting of a mixture of biosolids with an eroded soil, at the morphological and physiological level and the synthesis of some antioxidant compounds. The eroded soil was selected because, as mentioned above, in the Moldavian Plateau, soil erosion affects a large part of the arable land.

## 2. Results

### 2.1. Plant Morpholgy

Table 1 shows the morphological parameters of basil. In general, the morphological parameters were affected by the eroded soil and the addition of biosolids determined an improved growth even compared to the control. Thus, the fresh biomass had the lowest value in S3 eroded soil 17 g, 35% lower compared to S4 control. At S1 biosolids, the biomass had values close to S4, while at S2, the mixture substrate had the highest biomass of 42 g, 140% higher than S3 and 54% higher than S4. On the other hand, the dry mass recorded higher values compared to the control in all variants, with increases of up to 35% in S1 compared to S4. Regarding plant height, the lowest value was recorded at S3 38 cm, 5% lower than S4, while the highest value was recorded at S2 43 cm, 8% higher than S4; however, the differences were statistically insignificant. The number of stems was the lowest in S3, 26% lower than S4, while the highest value was recorded in the mixture substrate, 47% higher than S3.

### 2.2. Plant Physiology

**Figure 1:**
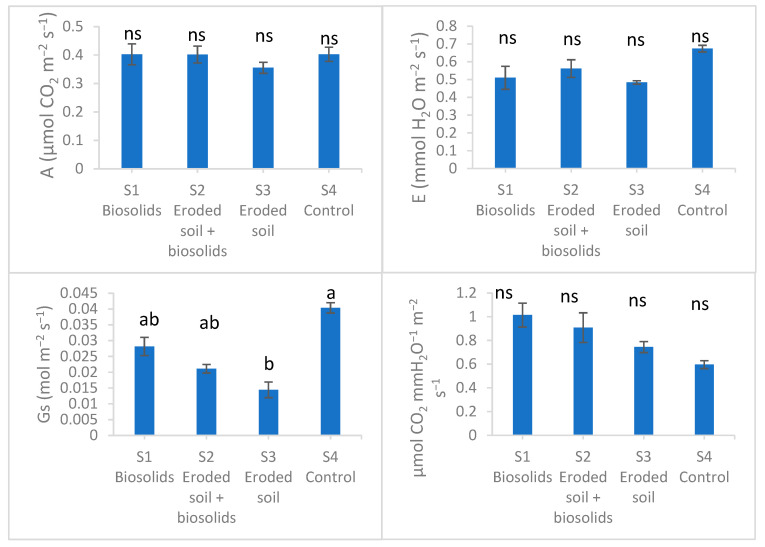
Parameters of gas exchange in basil grown on experimental substrates with eroded soil and biosolids. A—presynthesis rate, E—transpiration rate, Gs—stomatal conductance, A/E water use efficiency. Values are means with standard errors. N = 27. Different lowercase letters mean significant differences Tukey Test comfort for *p* ˂ 0.05. ns—not significant.

The gas exchange parameters are shown in Figure 1. The rate of photosynthesis and the rate of transpiration decreased in all experimental substrates, the greatest decreases being recorded in S3 eroded soil by 11 and 28%, respectively, compared to S4 control, while the values from S1 and S2 were very close to those of the control, the differences not being significant. Furthermore, the stomatal conductance decreased in the experimental substrates, significantly, by up to 64% in S3. On the other hand, the water use efficiency values were higher in the experimental substrates compared to the control, but the differences were not significant.

Table 2 shows the parameters of chlorophyll fluorescence in light-adapted basil leaves. The quantum yield (ΦPSII) of PSII significantly increased by 6% at S2 compared to the S4 control. On the other hand, initial or stable fluorescence (Fs) and the maximum fluorescence (Fm′) recorded the lowest values also at S2, with a significant decrease of 76% and 70% compared to the control.

The content of chlorophyll and carotenoids is shown in Table 3. Although no significant differences were recorded, an increase in the content of chlorophyll a, b, and carotenoids was found in S1, by 6, 28, and 14% compared to Control. On the other hand, the content of chlorophyll a and carotenoids recorded the lowest values at S3, with decreases of 17 and 4% compared to the control. The ratio of chlorophyll a/b decreased in all experimental substrates compared to the control.

**Figure 2:**
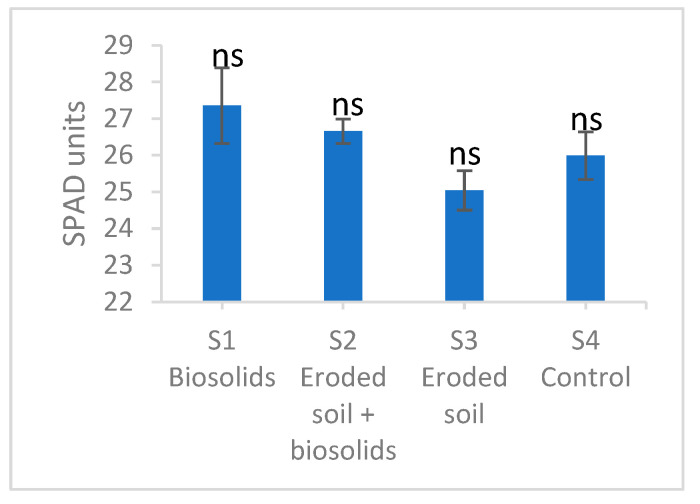
Total chlorophyll content in basil grown on experimental substrates with eroded soil and biosolids. Values are means with standard errors. N = 27. ns—not significant according to Tukey Test for *p* ˂ 0.05.

The total chlorophyll content determined with the SPAD meter is shown in Figure 2. Although the differences are not significant, a slight decrease in S3 and a slight increase in S1 compared to the control can be observed.

The activity of antioxidant enzymes and soluble protein content are presented in Table 4. Catalase activity was significantly higher in all experimental substrate variants, with increases of 68%, 105%, and 137% in S1, S2, and S3 compared to the control. Regarding the activity of peroxidase and superoxide dismutase, the values increased in all the experimental substrates compared to the control, but the differences were not significant. The soluble protein content was also higher in the experimental variants, but the differences were not significant.

**Figure 3:**
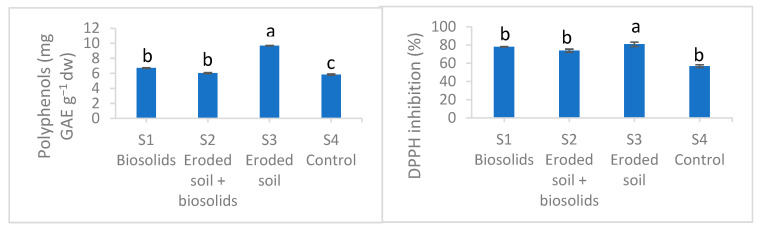
Polyphenols content and antioxidant activity of basil grown on experimental substrates with eroded soil and biosolids. Values are means with standard errors. N = 3. Different lowercase letters mean significant differences Tukey Test comfort for *p* ˂ 0.05. ns—not significant.

The total content of polyphenols and the antioxidant activity of basil extracts are shown in Figure 3. The content of polyphenols was significantly higher in all experimental substrate variants, with increases of 15, 3, and 66% in S1, S2, and S3 compared to Control. Regarding the antioxidant activity, the values were higher in all the experimental variants of the substrates, but only in S3 there was a significantly higher increase of 42% compared to the control.

The content of caffeic acid, rosmarinic acid and gallic acid is shown in Table 5. The content of caffeic acid was significantly higher in S2 and S3 by 183 and 66% compared to the control, while in S1 there was a reduction by 16% compared to the control; however, this was statistically insignificant. On the other hand, the rosmarinic acid content increased significantly in all the experimental substrates, by 154, 225, and 509% compared to the control. And the content of gallic acid increased in all the experimental substrates, by 44%, 188%, and 144% in S1, S2, and S3 compared to the control.

## 3. Discussion

Currently, agriculture based on chemical fertilization faces two big challenges. One is related to the production of gases with a greenhouse effect and water and air pollution when they are used in excess [16]. The other is related to the increase in price of fertilizers to record levels as well as the reduced availability on the market as a result of the decision of some large producing countries such as China to ban the export [17]. The use of sewage sludge for the fertilization of crops can be a sustainable solution in the context in which, worldwide, increasingly larger quantities are produced [18]. In order to be used in agriculture, sewage sludge must meet legislative norms. In Romania, MO 344/2004 regulates the maximum limits of heavy metals from sewage sludge, from the soil on which it is to be administered and their concentration for a period of 10 years. The maximum allowable concentrations of heavy metals in sludge intended for use in agriculture (mg/kg of dry matter): Cd 10, Cu 500, Ni 100, Pb 300, Zn 2000, Hg 5, Cr 500, Co 50, As 10. In general, sewage sludge produced in Romania contains heavy metals, but far below the maximum limit allowed for use in agriculture. Balanica et al., 2018 [19] showed that the content of heavy metals in sludge from five sewage treatment plants from South of Romania Giurgiu, Calarasi, Braila, Galati and Tulcea is below the maximum allowed limit. Ailincai et al., 2012 [20] showed that the two-year application of 30 t/ha of sludge from the sewage treatment plant in Iasi on an arable land in the Moldavian Plateau contributed to an improvement in soil properties through an increase in the organic carbon content from soil by 4.6 g/kg (28%), compared to the unfertilized variant. Utilization of sewage sludge in agriculture is of significant interest, due to nitrogen and phosphorus and microelements supply, Zn, Cu, B, and Mn. In this study, sewage sludge from the Wastewater Treatment Plant in the city of Iasi, Romania was used. The results in this study showed that the fresh biomass increased in the basil grown on the mixed substrate. This positive effect on the growth of plants fertilized with biosolids can be triggered by the rich content of nutrients (N, P, K, Mg, Mn, Zn) used by plants in the primary metabolic processes of growth and development. Positive choices on production indicators were also reported in other species. For example, Cocarta et al., 2017 [21] showed that the fertilization of wheat with sewage sludge at a dose of 25 t/ha increases production, while the content of Cd and Pb in the soil and in the wheat were below the regulated limit. Regarding the effects at the physiological level in this study, a slight reduction in the activity of photosynthesis and the content of assimilatory pigments was found, simultaneously with an increase in the activity of the antioxidant enzymes SOD, POD, and CAT. This can be associated both with the reduced content of micro and macronutrients in the eroded soil, which limits the process of photosynthesis and therefore plant growth. Furthermore, the physical properties of the soil, such as the high degree of compaction and low water-retention capacity may also impair the normal physiological processes in plants. In the variant with mixed substrate, these negative effects were not observed, which may suggest that the addition of biosolids contributes both to supplementing the mineral elements necessary for plant growth and to improving the physical properties of the soil.

Plants are equipped with enzymatic and non-enzymatic protective systems against oxidative stress caused by heavy metals. For example, Antolin et al., 2010 [22] showed that the cultivation of alfalfa on sewage sludge led to the accumulation of heavy metals in the plant tissue, causing oxidative stress through the induction of the antioxidant enzymatic activities and alterations in the redox state of ascorbate. Regarding the synthesis of phenolic compounds and the antioxidant activity, a positive correlation was found between the total content of polyphenols and the antioxidant activity. Higher values of the content of total polyphenols were recorded in plants cultivated on eroded soil. In plants, polyphenols are products of secondary metabolism with a defense role, which demonstrates the fact that eroded soil induces a strong stress on basil. In this study, the content of caffeic acid, rosmarinic acid and gallic acid in the basil leaf extract was measured. The three compounds are phenolic compounds found in various plants and fruits used in the human diet. Specifically, rosmarinic acid is the phenolic acid found in the largest amount in basil. All three studied compounds have multiple applications such as preservatives in the food industry, cosmetic and pharmaceutical industry [23,24]. Also, these compounds have numerous bioactivities such as antioxidant, cardio protective role, antibacterial activity [25], antiviral activity, anti-inflammatory activity, anti-anticancer activity, and antihepato-cellular carcinoma activity [26]. The values obtained in this study are close to those presented in the literature [27] and pertains to the opportunity of using basil as a potential specie for the remediation of specific soil conditions, as shown with other common species for different environments [28]. Although the application of biosolids led to a lower synthesis of bioactive compounds, the positive effects at the morphological and physiological level support its use on soils affected by erosion for the cultivation of basil. The integration of sewage sludge into agricultural practices not only enhances soil fertility and nutrient content but also aligns with sustainable agricultural principles, reducing dependence on conventional chemical fertilizers [29]. Despite its potential benefits, the utilization of biosolids in agriculture remains relatively low in regions such as Romania. This disparity is influenced by both public perception and the stringent regulatory norms governing the use of biosolids [30]. As the field continues to evolve, future research endeavors should delve into regional dynamics, considering the unique challenges and opportunities associated with sewage sludge application in specific locations. In the Romanian context, a nuanced understanding of public concerns, coupled with tailored research on crop-specific responses, could provide valuable insights for refining guidelines and encouraging more widespread and sustainable utilization of biosolids in agriculture.

## 4. Materials and Methods

### 4.1. Growing Plants in Laboratory Conditions

Sweet basil seeds *Ocimum basilicum* L. obtained from the Station for Vegetables Research-Development at Buzau, Romania (https://scdlbuzau.ro/ (accessed on 1 November 2023)) were used in this study. The tested cultivar ‘Aromat de Buzau’ has green leaves and white flowers and the predominant compounds in the essential oil that give it its aroma are linalool and metyl chavicol. This species was selected because, besides its rich content of essential oil, it contains numerous valuables phytochemical compounds including phenolic acids. Cultivating medicinal plants for their secondary products (phenolic acids) eliminates the hypothetical risk of human contamination with pollutants in case on plants fertilized with biosolids. 

The substrates tested were the biosolids from the wastewater treatment plant in Iasi (47.1495, 27.6623) and the eroded soil (loamy chernozem) taken from an arable land in the Moldavian Plateau (46.2029, 27.4144). Biosolids are produced through the secondary treatment of wastewater sludge. Secondary treatment is achieved through biological treatment to reduce the nitrogen content, the addition of a composite for dehydration and thickening, and drying on an open-air platform. The content of heavy metals was below the maximum allowable limits for agricultural use: Co (2.07 mg/kg), Ni (9.38 mg/kg), Cu (32.07 mg/kg), Zn (952.1 mg/kg), As (7.09 mg/kg), Cd (0.55 mg/kg), Hg (0.18 mg/kg), Pb (1.5 mg/kg), Al (271 mg/kg), Cr (27.0 mg/kg). Four experimental variants were created and tested: S1 biosolids 100%, S2 biosolids 15% + eroded soil 85%, S3 eroded soil 100% and S4 control (commercial growing substrate). 

The biosolids were characterized by a high content of organic matter (26.6%) and nutrients (total N 4.83%, P 2.18%, and K 0.46%), while the pH was 6.96 and the electrical conductivity 6280 µS/cm. The eroded soil was a loamy chernozem composed of coarse sand 3.5%, medium sand 45.4%, fine sand 22.8%, clay 28.3%, and physical clay 37.3%. The soil was characterized by a reduced content of organic matter (1.24%) and nutrients (total N 0.068%, P 0.008%, and K 0.02%), while the pH was 8.05 and the electrical conductivity 221.4 µS/cm. All the investigated physicochemical characteristics were improved in the mixed substrate as a result of the biosolids addition (organic matter 20.5%, total N 3.7%, P 0.91%, and K 0.29%, pH 7.5, and the electrical conductivity 5200 µS/cm. According to the manufacturer’s specifications, the commercial growing substrate that was used as a control, (Florisol, Botosani, Romania), has an organic matter content of at least 70%, N 410 ppm, P 192 ppm, K 1350 ppm, pH 6.5–7.0, and humidity 60–70%.

Black plastic pots with a volume of one liter were used. The pots were filled with the experimental substrates, nine pots for each variant, and the basil seeds (five/pot) were put to germination on 7th of April directly in these pots. After one week, the plants were thinned to one per each pot. The pots were watered daily each with 50 mL of distilled water. Lighting was done with fluorescent tubes of 18 W, 4500 k for ten hours a day. The temperature was 24 °C during the day and 22 °C at night. The relative humidity was 60%. The plants were harvested on 7th of July when the plants reached flowering.

### 4.2. Morphological Measurements

After harvesting, the plants were weighed to determine the fresh biomass, and then the height of the plants was measured with a ruler and the lateral stems were counted. The dry weight of the plants was determined by drying the plants in an oven at 105 °C until the weights were stabilized.

### 4.3. Physiological Measurements

One day before harvesting plant photosynthesis, chlorophyll fluorescence, total con-tent of chlorophyll, and the assimilatory pigments chlorophyll a, b and carotenoids were measured.

Photosynthesis (µmol m^−2^ s^−1^) was measured with the LCi system (ADC Bioscientific, Hoddesdon, UK) equipped with a broad leaf chamber with an area of 6.4 cm^2^.

Chlorophyll fluorescence parameter ΦPSII (quantum yield of PSII of light-adapted leaves) was measured with FMS2 (Hansa Tech Ltd., Hoddesdon, UK).

The total chlorophyll content was measured with SPAD 502 (Konica Minolta, Osaka, Japan) and expressed in SPAD units.

The assimilatory pigments (chlorophyll a, b and carotenoids) were quantified by extracting 0.1 g of the leaf in 80% acetone and reading the absorbance at 470, 646, and 663 nm [31] (Wellburn, 1994).

### 4.4. Biochemical Measurements

The activity of the antioxidant enzymes CAT (EC 1.11.1.6), SOD (EC 1.15.1.1), POD (EC 1.11.1.7), were measured according to the methods described in Liu et al., [32] and results were expressed as enzyme unit/mg protein. The soluble protein content was measured according to Bradford (1976) [33] and the results were expressed as mg/g fw.

The total content of polyphenols and the antioxidant activity were quantified according to the methods described in Lobiuc et al., [34].

The content of caffeic, rosmarinic and gallic phenolic acids was determined by High Performance liquid chromatography (Shimadzu LC-10ADVP, Columbia, MD, USA) coupled to a photodiode array detector (Shimadzu SPD-M20A, Columbia, MD, USA) and a C18 (Macherey-Nagel) reverse phase (150 mm × 4.6 mm × 4 µm) column. The mobile phases were water—acetic acid (99:1) (A) and methanol (B), the flow rate was 0.6 mL/min temperature 40 °C. The program was 0 min 100% A, 5 min 6% B, 5–7 min 6% B, 50 min 30% B, 50–52 min 30% B, 62 min 100% B. The individual phenolic acids were quantified using external standards, HPLC grade (Sigma, Steinheim, Germany).

### 4.5. Statistical Analysis

The data were processed with EXCEL and SPSS v21 programs. The means and standard errors (S.E.) were calculated and the results were expressed as mean ± S.E. The data were checked for normality using the Shapiro–Wilk test. Furthermore, because the data were normally distributed, the significance of the differences between treatments could be tested using ANOVA single factor followed by the Tukey multiple comparation Test for *p* ˂ 0.05.

## 5. Conclusions

The cultivation of basil on eroded soil produced negative changes manifested by the reduction in plant height, number of side branches, and finally the biomass. The addition of biosolids led to the removal of these negative effects, with the plants performing even better than the control plants. Negative effects of cultivation on the eroded soil were also recorded at the physiological level. These effects were manifested by a slight reduction in the rate of photosynthesis and the content of assimilatory pigments, but also an increase in oxidative stress. The addition of biosolids also led to an improvement in physiological functions. Regarding the synthesis of bioactive compounds, total polyphenols and phenolic acids (caffeic, rosmarinic, and gallic), an increase was found in plants grown on eroded soil. This preliminary study shows the positive effects of treating soils affected by erosion with biosolids. At the same time, the fact that the plants also grew on 100% biosolids shows the possibility of cultivating these species directly on the biosolids in order to improve its quality before agricultural use.

## Figures and Tables

**Table 1:** Morphological parameters of basil grown on experimental substrates with eroded soil and biosolids.

Substrate	Fresh Mass (g)	Dry Mass (%)	Height (cm)	Number of Stems (No.)
S1 Biosolids	28.5 ± 0.80 b	11.4 ± 0.18 a	42.8 ± 4.38 ns	18.0 ± 2.24 a
S2 Eroded soil + biosolids	42.0 ± 0.87 a	9.66 ± 0.22 ab	43.3 ± 1.97 ns	18.7 ± 0.10 a
S3 Eroded soil	17.5 ± 0.65 c	9.19 ± 0.25 b	38.0 ± 1.51 ns	12.7 ± 0.53 ab
S4 Control	27.2 ± 0.41 b	8.42 ± 0.19 c	40.1 ± 1.73 ns	17.2 ± 0.37 b

Values are means with standard errors. N = 8. Different lowercase letters mean significant differences Tukey Test comfort for *p* ˂ 0.05. ns—not significant.

**Table 2:** Actual PSII quantum yield in basil grown on eroded soil and biosolids.

Substrate	Fs	Fm′	ΦPSII
S1 Biosolids	403 ± 15.92 b	2083 ± 72.41 a	0.810 ± 0.01 b
S2 Eroded soil + biosolids	103 ± 9.63 c	581 ± 43.98 c	0.820 ± 0.00 a
S3 Eroded soil	447 ± 17.38 a	1954 ± 68.51 b	0.770 ± 0.01 c
S4 Control	367 ± 21.55 bc	1738 ± 98.61 bc	0.790 ± 0.00 bc

Values are means with standard errors. N = 8. Different lowercase letters mean significant differences Tukey Test comfort for *p* ˂ 0.05.

**Table 3:** Chlorophyll and carotenoids content in basil grown on experimental substrates with eroded soil and biosolids.

Substrate	Chlorophyll a (mg/g fw)	Chlorophyll b (mg/g fw)	Carotenoids a (mg/g fw)	Chlorophyll a/b (mg/g fw)
S1 Biosolids	1.20 ± 0.12 ns	0.540 ± 0.06 ns	0.220 ± 0.01 ns	2.620 ± 0.42 ns
S2 Eroded soil + biosolids	1.16 ± 0.03 ns	0.460 ± 0.06 ns	0.210 ± 0.01 ns	2.220 ± 0.02 ns
S3 Eroded soil	0.930 ± 0.11 ns	0.450 ± 0.04 ns	0.190 ± 0.01 ns	2.08 ± 0.05 ns
S4 Control	1.12 ± 0.13 ns	0.420 ± 0.13 ns	0.200 ± 0.05 ns	3.06 ± 0.61 ns

Values are means with standard errors. N = 8. Different lowercase letters mean significant differences Tukey Test comfort for *p* ˂ 0.05. ns—not significant.

**Table 4:** Antioxidant enzyme activity and soluble proteins content in basil grown on experimental substrates with eroded soil and biosolids.

Substrate	Catalase (Units mg^−1^ Protein)	Peroxidase (Units mg^−1^ Protein)	Superoxide Dismutase (Units mg^−1^ Protein)	Soluble Protein (mg g^−1^ fw)
S1 Biosolids	182 ± 6.87 b	3.93 ± 0.35 ns	3.99 ± 0.81 ns	9.01 ± 0.13 ns
S2 Eroded soil + biosolids	223 ± 10.93 a	3.84 ± 0.37 ns	4.07 ± 0.12 ns	10.1 ± 0.29 ns
S3 Eroded soil	257 ± 7.13 a	3.73 ± 0.77 ns	5.41 ± 1.48 ns	9.39 ± 0.15 ns
S4 Control	108 ± 5.77 c	2.81 ± 0.37 ns	3.63 ± 0.47 ns	9.50 ± 0.45 ns

Values are means with standard errors. N = 3. Different lowercase letters mean significant differences Tukey Test comfort for *p* ˂ 0.05. ns—not significant.

**Table 5:** Phenolic acids content in basil grown on experimental substrates with eroded soil and biosolids.

Substrate	Caffeic Acid (mg g^−1^ dw)	Rosmarinic Acid (mg g^−1^ dw)	Galic Acid (mg g^−1^ dw)
S1 Biosolids	0.25 ± 0.01 c	8.69 ± 0.41 c	0.13 ± 0.00 bc
S2 Eroded soil + biosolids	0.85 ± 0.00 a	11.09 ± 0.05 b	0.26 ± 0.00 a
S3 Eroded soil	0.50 ± 0.03 b	20.77 ± 0.04 a	0.22 ± 0.04 ab
S4 Control	0.30 ± 0.01 c	3.41 ± 0.01 d	0.09 ± 0.01 c

Values are means with standard errors. N = 8. Different lowercase letters mean significant differences Tukey Test comfort for *p* ˂ 0.05.

## Data Availability

All data generated in this study are presented in the article.
